# Supplementary material for: Insights into the Functional Responses of Four Neotropical-Native Parasitoids to Enhance Their Role as Biocontrol Agents Against Anastrepha fraterculus Pest Populations
Source: Insects. 2025 Sep 2;16(9):919. doi: 10.3390/insects16090919 (PMC12471003; doi:10.3390/insects16090919)
Supplement: Supplementary file 1 [file insects-16-00919-s001.zip › Nuñez-Campero et al. Author profiles.pdf]

Author Profiles

Segundo Núñez-Campero, PhD in Biological Sciences and researcher at the La Rioja Regional Center for Scientific Research and Technology Transfer (CRILAR-CONICET). His expertise encompasses insect biology and ecology, species distribution modeling using GIS, and experimental design. Currently, his research focuses on Tephritidae fruit flies and their parasitoids, with previous work on Neuroptera diversity and biology for biological control. Member of the Argentine Entomological Society (SEA). segundo.nc@conicet.gov.ar. ORCID: 0000-0003-0382-4155

Lorena del Carmen Suárez, biologist by profession; PhD from the National University of Cuyo (Argentina). Junior Research Scientist at CONICET (National Council for Scientific and Technical Research from Argentina). I work at the Plant, Animal and Food Health Department of the Agricultural, Livestock and Agroindustry Secretariat (Government of the Province of San Juan, Argentina). My research topic covers the integrated management of agricultural insect pests, particularly eco-friendly pest control methods. I am currently working on biological control, mass trapping and sterile insect techniques for fruit fly control in Argentina. Most of my scientific papers involve studies on biology, ecology and mass rearing of parasitoids associated with fruit fly pests; biological control of those pests, and assessment of natural attractants for trapping the global pest Ceratitis capitata. Coordinator of mass rearing production of medfly and parasitoids at San Juan Biofactory, Argentina. The h-index is 8 to date (March, 2025). lorenasuarez@conicet.gov.ar. Scopus ID: 55578753100, ORCID: 0000-0002-4341-8189

Flávio Roberto Mello Garcia, an entomologist with a master's and a doctorate in Zoology from the Pontifical Catholic University of Rio Grande do Sul (Brazil), is a professor at the Federal University of Pelotas (Brazil) and a visiting professor at the University of Florida and USDA (USA). His influence extends beyond his research, as he has mentored students from the United States, Haiti, Brazil, Italy, Mozambique, Peru, Portugal, and Uruguay, nurturing the next generation of entomologists and inspiring hope for the future of the field. His research, which is at the forefront of the field, focuses on managing Drosophila suzukii (Diptera: Drosophilidae) and fruit flies (Diptera: Tephritidae), emphasizing biological control using parasitoids and entomopathogenic nematodes. He is the coordinator of the first biological control project using parasitoids and sterile insect techniques to control Drosophila suzukii in South America. He is responsible for the first patent for a non-toxic product in the control of Drosophila suzukii in South America. flavio.garcia@ufpel.edu.br. ORCID: 0000-0003-0493-1788

Jorge Cancino. Holds a Master of Science in Biology from the National Autonomous University of Mexico (UNAM) and a Doctorate (PhD) from El Colegio de la Frontera Sur (ECOSUR). He has more than 30 years of experience in mass rearing, packing, and

releasing fruit fly parasitoids in the National Fruit Fly Program (SENASICA-SADER). He currently works as a professor and researcher at the Autonomous University of Chiapas (UNACH), where he develops projects on biological control of various tropical pests. He is a member of the National System of Researchers (SECIHTI) with the title of National Researcher Level II. jorge.cancino@unach.mx, ORCID: 0000-0003-3287-3060

Pablo Montoya holds a BSc (1983) and PhD (1999) in Biological Sciences from the National Autonomous University of Mexico (UNAM). For 29 years he was the research leader in the Moscamed and Moscafrut programs of SENASICA-SADER in Mexico. His main research lines focus on the biology and ecology of natural enemies with potential to be used as biological control agents against fruit flies and other agricultural pests, the development of agricultural pest detection and other control systems, as well as the development and application of the Sterile Insect Technique (SIT). He currently works as a professor at the Institute of Biosciences of the Autonomous University of Chiapas, specializing in Ecology, Biodiversity, and Sustainable Development, as well as a Visiting Researcher at the Colegio de la Frontera Sur (ECOSUR) in Tapachula, Chiapas, México. He has been a member of the National System of Researchers (SECIHTI) since 2001 and currently holds the title of National Researcher Level III. pablo.montoya@unach.mx. His h-index is 26 as of March 2025; Scopus ID: 7005501527; ORCID: 0000-0002-8415-3367.

Sergio Marcelo Ovruski. PhD in Biological Sciences graduated from National University of Tucumán (Argentina) in 1995, is working with economically important fruit flies and their hymenopterous parasitoids since 1998 as researcher of the National Council of Science and Technology of Argentina (CONICET in Spanish). He currently is a CONICET Senior Researcher at the Pilot Plant of Industrial Microbiological Processes and Biotechnology (PROIMI in Spanish) in Tucumán, Argentina. He is the head of the Bioecoetological Research of Fruit Flies and their Natural Enemies Laboratory which belongs to the Pest Biological Control Department. His research topics involve biology, ecology, behavior and management via eco-friendly methods, mainly biological control. He has been director and co-director of numerous PhD theses, doctoral and postdoctoral fellows, junior researchers of CONICET, national and international research projects and technological development and research agreements between CONICET and provincial governments from Argentina. Past fellow of the International Atomic Energy Agency. Coordinator of mass rearing production of medfly and parasitoids at San Juan Biofactory, Argentina. His h-index is 22 to date (March 2025). sovruski@conicet.gov.ar. Scopus ID: 6603080215, ORCID number: 0000-0002-0137-4499.
