# Supplementary material for: Insights into the Functional Responses of Four Neotropical-Native Parasitoids to Enhance Their Role as Biocontrol Agents Against Anastrepha fraterculus Pest Populations
Source: Insects. 2025 Sep 2;16(9):919. doi: 10.3390/insects16090919 (PMC12471003; doi:10.3390/insects16090919)
Supplement: Supplementary file 1 [file insects-16-00919-s001.zip › Nuñez-Campero et al. File_S2 Full table with discarded and proportion of hosts attacked.pdf]

Supplemental material S2. Expected number of hosts discarded (*De*) and expected proportion (P_d) of hosts attacked at each initial density of host (N_0) for the parasitoids *Coptera haywardi*, *Doryctobracon crawfordi*, *Ganaspis pelleranoi*, and *Opius bellus*.

N_0	*Parasitoid species*							
	C. haywardi		*D. crawfordi*		*G. pelleranoi*		*O. bellus*	
	De	P_d	***De***	P_d	***De***	P_d	***De***	P_d
1	0.25	0.25	0.85	0.85	0.73	0.72	0.78	0.77
5	1.38	0.27	4.08	0.81	3.20	0.64	4.07	0.81
10	4.75	0.47	8.18	0.81	6.12	0.61	8.47	0.84
15	9.19	0.61	12.44	0.82	9.06	0.60	13.06	0.87
20	13.94	0.69	16.83	0.84	12.11	0.60	17.76	0.88
40	33.64	0.84	35.34	0.88	25.69	0.64	37.11	0.92
60	53.56	0.89	54.60	0.91	41.26	0.68	56.81	0.94
80	73.53	0.91	74.18	0.92	58.23	0.72	76.64	0.95
100	93.51	0.93	93.91	0.93	76.09	0.76	96.52	0.96
