# Supplementary material for: Insights into the Functional Responses of Four Neotropical-Native Parasitoids to Enhance Their Role as Biocontrol Agents Against Anastrepha fraterculus Pest Populations
Source: Insects. 2025 Sep 2;16(9):919. doi: 10.3390/insects16090919 (PMC12471003; doi:10.3390/insects16090919)
Supplement: Supplementary file 1 [file insects-16-00919-s001.zip › Nuñez-Campero et al.,File_S1 The step-by-step codes for the package “frair” in R.pdf]

The functional response of four Neotropical fruit fly parasitoids and rearing process optimization

Núñez Campero, S.R., Ovruski, S.M.

2022-10-21

Data preparation, R libraries, auxiliary functions

This document was created as a modification from (Pritchard et al. 2017)

```
library(readxl)
rf <- read_excel("rf.xlsx")
View(rf)
attach(rf)

library(frair)
```

```
## Loading required package: stats4
```

```
## Loading required package: bbmle
```

```
library(RColorBrewer)
library(TeXCheckR)
library(knitr)
library(magrittr)
library(kableExtra)

ch <- rf[rf$spp=="Ch",]
Ob <- rf[rf$spp=="Ob",]
Dc <- rf[rf$spp=="Dc",]
Gp <- rf[rf$spp=="Gp",]


### Auxiliar function (mtexti & do_plot)

mtexti <- function(text, side, off = 0.12, srt = NULL, ...) {
  if(is.null(srt)){
    srt <- if(side == 2) 90 else
      if(side == 4) 270 else
        0
  }
  # dimensions of plotting region in user units
  usr <- par('usr')
  # dimensions of plotting region in inches
  pin <- par('pin')
  # user units per inch
  upi <- c(usr[2]-usr[1],
           usr[4]-usr[3]) / pin
```

```
  # default x and y positions
  xpos <- (usr[1] + usr[2])/2
  ypos <- (usr[3] + usr[4])/2
  if(1 == side)
    ypos <- usr[3] - upi[2] * off
  if(2 == side)
    xpos <- usr[1] - upi[1] * off
  if(3 == side)
    ypos <- usr[4] + upi[2] * off
  if(4 == side)
    xpos <- usr[2] + upi[1] * off
  text(x=xpos, y=ypos, text, xpd=NA, srt=srt, ...)
}

## Do general plot
do_plot <- function(x_axis_labs = FALSE, leg = 1){
  data("rf")
  chc <- brewer.pal(6, "Dark2")[1]
  Dcc <- brewer.pal(6, "Dark2")[2]
  Gpc <- brewer.pal(6, "Dark2")[3]
  Obc <- brewer.pal(6, "Dark2")[6]
  with(rf, plot(dens, par, type='n', ann = F, axes = F, xlim=c(0,101)))
  box(); axis(1, lwd = 0, lwd.ticks = 1); axis(2, lwd = 0, lwd.ticks = 1)
  with(ch, points(dens-0.5, par, pch=20, col=adjustcolor(chc, alpha = 1)))
  with(Dc, points(dens+0.5, par, pch=20, col=adjustcolor(Dcc, alpha = 1)))
  with(Gp, points(dens-1, par, pch=20, col=adjustcolor(Gpc, alpha = 1)))
  with(Ob, points(dens+1, par, pch=20, col=adjustcolor(Obc, alpha = 1)))
  if(as.logical(leg)){
    if(leg == 1){
      text <- c(substitute(paste(italic('C. haywardi'))), substitute(
          paste(italic('D. crawfordi'))), substitute(
            paste(italic('G. pelleranoi'))), substitute(
              paste(italic('O. bellus'))))
      pch <- 20
      col <- c(chc, Dcc, Gpc, Obc)
      lty <- NULL
    } else if(leg == 2){
      text <- c(substitute(
        paste(italic('C. haywardi'))), substitute(
          paste(italic('D. crawfordi'))), substitute(
            paste(italic('G. pelleranoi'))), substitute(
              paste(italic('O. bellus'))), 'Reasonable Values')
      pch <- c(20, 20, 20, 20, NA)
      col <- c(chc, Dcc, Gpc, Obc, "grey25")
      lty <- c(NA, NA, NA, NA, 2)
    } else if(leg == 3){
      text <- c(substitute(
        paste(italic('C. haywardi'))), substitute(
          paste(italic('D. crawfordi'))), substitute(
            paste(italic('G. pelleranoi'))), substitute(
              paste(italic('O. bellus'))), 'Reasonable Values', "Fitted Curve")
      pch <- c(20, 20, 20, 20, NA, NA)
      col <- c(chc, Dcc, Gpc, Obc, "grey25", "grey25")
```

```r
      lty <- c(NA, NA, NA, NA, 2, 1)
    }
    legend(0, 45, legend = text, pch = pch, col=col, lty=lty, bty='n')
  }
  mtext('N° host attacked', 2, line=2.5, las=0)
  if(x_axis_labs){
    mtext('Host Density', 1, line=2.5, las=0)
  }
}

### Do plot C. haywardi

do_Ch_plot <- function(x_axis_labs = FALSE, leg = 1){
  data("ch")
  chc1 <- brewer.pal(6, "Dark2")[1]
  with(ch, plot(dens, par, type='n', ann = F, axes = F, xlim=c(0,101)))
  box(); axis(1, lwd = 0, lwd.ticks = 1); axis(2, lwd = 0, lwd.ticks = 1)
  with(ch, points(dens-0.5, par, pch=20, col=adjustcolor(chc1, alpha = 1)))
  if(as.logical(leg)){
    if(leg == 1){
      text <- c(substitute(paste(italic('C. haywardi'))))
      pch <- NA_integer_
      col <- c(chc1)
      lty <- NULL
    } else if(leg == 2){
      text <- c(substitute(
        paste(italic('C. haywardi'))), 'Reasonable Values')
      pch <- c(20, NA)
      col <- c(chc1, "grey25")
      lty <- c(NA, 2)
    } else if(leg == 3){
      text <- c(substitute(
        paste(italic('C. haywardi'))), 'Reasonable Values', "Fitted Curve")
      pch <- c(20, NA, NA)
      col <- c(chc1,"grey25", "grey25")
      lty <- c(NA, 2, 1)
    }
    legend(0, 20, legend = text, pch = pch, col=col, lty=lty, bty='n')
  }
  mtext('N° host attacked', 2, line=2.5, las=0)
  if(x_axis_labs){
    mtext('Host Density', 1, line=2.5, las=0)
  }
}

### Do plot D. crawfordi

do_Dc_plot <- function(x_axis_labs = FALSE, leg = 1){
  data("Dc")
  Dcc1 <- brewer.pal(6, "Dark2")[1]
  with(Dc, plot(dens, par, type='n', ann = F, axes = F, xlim=c(0,101)))
  box(); axis(1, lwd = 0, lwd.ticks = 1); axis(2, lwd = 0, lwd.ticks = 1)
  with(Dc, points(dens-0.5, par, pch=20, col=adjustcolor(Dcc1, alpha = 1)))
```

```r
  if(as.logical(leg)){
    if(leg == 1){
      text <- c(substitute(paste(italic('D. crawfordi'))))
      pch <- NA_integer_
      col <- c(Dcc1)
      lty <- NULL
    } else if(leg == 2){
      text <- c(substitute(
        paste(italic('D. crawfordi'))), 'Reasonable Values')
      pch <- c(20, NA)
      col <- c(Dcc1, "grey25")
      lty <- c(NA, 2)
    } else if(leg == 3){
      text <- c(substitute(
        paste(italic('D. crawfordi'))), 'Reasonable Values', "Fitted Curve")
      pch <- c(20, NA, NA)
      col <- c(Dcc1,"grey25", "grey25")
      lty <- c(NA, 2, 1)
    }
    legend(0, 30, legend = text, pch = pch, col=col, lty=lty, bty='n')
  }
  mtext('N° host attacked', 2, line=2.5, las=0)
  if(x_axis_labs){
    mtext('Host Density', 1, line=2.5, las=0)
  }
}

# Do plot O. bellus

do_Ob_plot <- function(x_axis_labs = FALSE, leg = 1){
  data("Ob")
  Obc1 <- brewer.pal(6, "Dark2")[1]
  with(Ob, plot(dens, par, type='n', ann = F, axes = F, xlim=c(0,101)))
  box(); axis(1, lwd = 0, lwd.ticks = 1); axis(2, lwd = 0, lwd.ticks = 1)
  with(Ob, points(dens-0.5, par, pch=20, col=adjustcolor(Obc1, alpha = 1)))
  if(as.logical(leg)){
    if(leg == 1){
      text <- c(substitute(paste(italic('O. bellus'))))
      pch <- NA_integer_
      col <- c(Obc1)
      lty <- NULL
    } else if(leg == 2){
      text <- c(substitute(
        paste(italic('O. bellus'))), 'Reasonable Values')
      pch <- c(20, NA)
      col <- c(Obc1, "grey25")
      lty <- c(NA, 2)
    } else if(leg == 3){
      text <- c(substitute(
        paste(italic('O. bellus'))), 'Reasonable Values', "Fitted Curve")
      pch <- c(20, NA, NA)
      col <- c(Obc1,"grey25", "grey25")
      lty <- c(NA, 2, 1)
```

```
    }
    legend(0, 15, legend = text, pch = pch, col=col, lty=lty, bty='n')
  }
  mtext('N° host attacked', 2, line=2.5, las=0)
  if(x_axis_labs){
    mtext('Host Density', 1, line=2.5, las=0)
  }
}

# Do plot G. pelleranoi

do_Gp_plot <- function(x_axis_labs = FALSE, leg = 1){
  data("Gp")
  Gpc1 <- brewer.pal(6, "Dark2")[1]
  with(Gp, plot(dens, par, type='n', ann = F, axes = F, xlim=c(0,101)))
  box(); axis(1, lwd = 0, lwd.ticks = 1); axis(2, lwd = 0, lwd.ticks = 1)
  with(Gp, points(dens-0.5, par, pch=20, col=adjustcolor(Gpc1, alpha = 1)))
  if(as.logical(leg)){
    if(leg == 1){
      text <- c(substitute(paste(italic('G. pelleranoi'))))
      pch <- NA_integer_
      col <- c(Gpc1)
      lty <- NULL
    } else if(leg == 2){
      text <- c(substitute(
        paste(italic('G. pelleranoi'))), 'Reasonable Values')
      pch <- c(20, NA)
      col <- c(Gpc1, "grey25")
      lty <- c(NA, 2)
    } else if(leg == 3){
      text <- c(substitute(
        paste(italic('G. pelleranoi'))), 'Reasonable Values', "Fitted Curve")
      pch <- c(20, NA, NA)
      col <- c(Gpc1,"grey25", "grey25")
      lty <- c(NA, 2, 1)
    }
    legend(0, 45, legend = text, pch = pch, col=col, lty=lty, bty='n')
  }
  mtext('N° host attacked', 2, line=2.5, las=0)
  if(x_axis_labs){
    mtext('Host Density', 1, line=2.5, las=0)
  }
}
```

MODEL SELECTION: Functional response test

```
frair_test(formula = par~dens, data = ch)
```

```
## FUNCTIONAL RESPONSE TEST
##
## Evidence for type-II response:   Yes
## Evidence for type-III response:  -
```

```
##
## Type-II logistic regression output:
##           Estimate Std. Error z value  Pr(>|z|)
## density -0.0270530  0.0011766 -22.992 < 2.2e-16 ***
## ---
## Signif. codes:  0 '***' 0.001 '**' 0.01 '*' 0.05 '.' 0.1 ' ' 1
```

```
frair_test(formula = par~dens, data = Ob)
```

```
## FUNCTIONAL RESPONSE TEST
##
## Evidence for type-II response:   Yes
## Evidence for type-III response:  -
##
## Type-II logistic regression output:
##           Estimate Std. Error z value  Pr(>|z|)
## density -0.0183412  0.0015022  -12.21 < 2.2e-16 ***
## ---
## Signif. codes:  0 '***' 0.001 '**' 0.01 '*' 0.05 '.' 0.1 ' ' 1
```

```
frair_test(formula = par~dens, data = Dc)
```

```
## FUNCTIONAL RESPONSE TEST
##
## Evidence for type-II response:   Yes
## Evidence for type-III response:  -
##
## Type-II logistic regression output:
##            Estimate  Std. Error z value  Pr(>|z|)
## density -0.01490074  0.00096952 -15.369 < 2.2e-16 ***
## ---
## Signif. codes:  0 '***' 0.001 '**' 0.01 '*' 0.05 '.' 0.1 ' ' 1
```

```
frair_test(formula = par~dens, data = Gp)
```

```
## FUNCTIONAL RESPONSE TEST
##
## Evidence for type-II response:   Yes
## Evidence for type-III response:  -
##
## Type-II logistic regression output:
##            Estimate  Std. Error z value  Pr(>|z|)
## density -0.01061357  0.00073009 -14.537 < 2.2e-16 ***
## ---
## Signif. codes:  0 '***' 0.001 '**' 0.01 '*' 0.05 '.' 0.1 ' ' 1
```

ALTERNATIVE METHOD:

This method fit a generalized model of functional response. Use the scalar exponent (q) parameterized as q= 0 representing the functional response type II.

Coptera haywardi

Variable "q"

```
ch_flex <- frair_fit(formula = par~dens, data = ch, response = "flexpnr", start =
    list(b = 1, h = 0.05, q = 0), fixed = list(T = 24/24))

summary(ch_flex$fit)
```

```
## Maximum likelihood estimation
##
## Call:
## bbmle::mle2(minuslogl = flexpnr_nll, start = start, method = "Nelder-Mead",
##     optimizer = "optim", fixed = fixed, data = list(X = dat$X,
##         Y = dat$Y), control = list(maxit = 5000))
##
## Coefficients:
##    Estimate Std. Error z value     Pr(z)
## b 1.5548816  0.4292719  3.6221 0.0002922 ***
## q 0.3858355  0.2077849  1.8569 0.0633256 .
## h 0.1529410  0.0066975 22.8357 < 2.2e-16 ***
## ---
## Signif. codes:  0 '***' 0.001 '**' 0.01 '*' 0.05 '.' 0.1 ' ' 1
##
## -2 log L: 1090.814
```

"q" fixed to zero:

```
ch_II <- frair_fit(formula = par~dens, data = ch, response = "flexpnr", start =
                     list(b = 1, h = 0.05), fixed = list(T = 24/24, q = 0))

summary(ch_II$fit)
```

```
## Maximum likelihood estimation
##
## Call:
## bbmle::mle2(minuslogl = flexpnr_nll, start = start, method = "Nelder-Mead",
##     optimizer = "optim", fixed = fixed, data = list(X = dat$X,
##         Y = dat$Y), control = list(maxit = 5000))
##
## Coefficients:
##   Estimate Std. Error z value     Pr(z)
## b 2.360667   0.334402  7.0594 1.673e-12 ***
## h 0.142983   0.005607 25.5008 < 2.2e-16 ***
## ---
## Signif. codes:  0 '***' 0.001 '**' 0.01 '*' 0.05 '.' 0.1 ' ' 1
##
## -2 log L: 1094.789
```

Model comparisons

```
AIC(ch_flex$fit, ch_II$fit)  #Corresponde Flex
```

```
##        AIC df
## 1 1096.814  3
```

```
## 2 1098.789  2
```

```r
AICtab(ch_flex$fit, ch_II$fit)
```

```
##              dAIC df
## ch_flex$fit 0    3
## ch_II$fit   2    2
```

Individual plot

```r
with(ch, plot(dens, par, main= c(substitute(paste(italic("C. haywardi")))), xlab = "Host density", ylab
x <- with(ch, seq(from= min(dens), to = max(dens), by = 1))
lines(x, flexpnr(X= x, b= 1.555, h= 0.153, q=0.386, T= 24/24), col="grey50", lty=1)
```

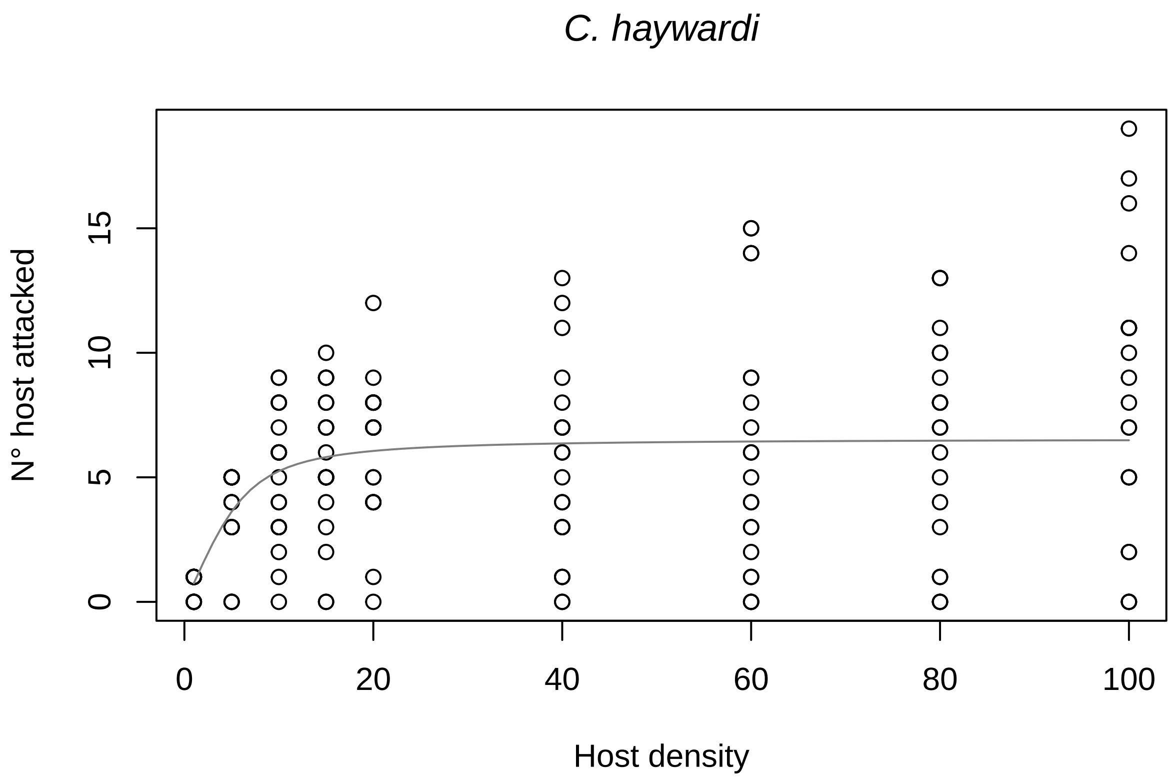

Doryctobracon crawfordi

Variable "q"

```r
Dc_flex <- frair_fit(formula = par~dens, data = Dc, response = "flexpnr", start =
                       list(b = 1, h = 0.03, q = 0), fixed = list(T = 24/24))

summary(Dc_flex$fit)
```

```
## Maximum likelihood estimation
##
## Call:
## bbmle::mle2(minuslogl = flexpnr_nll, start = start, method = "Nelder-Mead",
```

```
##     optimizer = "optim", fixed = fixed, data = list(X = dat$X,
##         Y = dat$Y), control = list(maxit = 5000))
##
## Coefficients:
##   Estimate Std. Error z value     Pr(z)
## b 0.177963   0.050040  3.5564 0.0003759 ***
## q 0.209688   0.125515  1.6706 0.0947949 .
## h 0.149761   0.012378 12.0990 < 2.2e-16 ***
## ---
## Signif. codes:  0 '***' 0.001 '**' 0.01 '*' 0.05 '.' 0.1 ' ' 1
##
## -2 log L: 3210.193
```

"q" fixed to zero:

```
Dc_II <- frair_fit(formula = par~dens, data = Dc, response = "flexpnr", start =
                     list(b = 1, h = 0.03), fixed = list(T = 24/24, q = 0))

summary(Dc_II$fit)
```

```
## Maximum likelihood estimation
##
## Call:
## bbmle::mle2(minuslogl = flexpnr_nll, start = start, method = "Nelder-Mead",
##     optimizer = "optim", fixed = fixed, data = list(X = dat$X,
##         Y = dat$Y), control = list(maxit = 5000))
##
## Coefficients:
##    Estimate Std. Error z value     Pr(z)
## b 0.2753474  0.0235731  11.681 < 2.2e-16 ***
## h 0.1295244  0.0084166  15.389 < 2.2e-16 ***
## ---
## Signif. codes:  0 '***' 0.001 '**' 0.01 '*' 0.05 '.' 0.1 ' ' 1
##
## -2 log L: 3213.234
```

Model comparison

```
AIC(Dc_flex$fit, Dc_II$fit)   ##Corresponde Flex
```

```
##        AIC df
## 1 3216.193  3
## 2 3217.234  2
```

```
AICtab(Dc_flex$fit, Dc_II$fit)
```

```
##             dAIC df
## Dc_flex$fit 0    3
## Dc_II$fit   1    2
```

Individual plot

```
with(Dc, plot(dens, par, main= c(substitute(paste(italic("D. crawfordi")))), xlab = "Host density", ylal
x <- with(Dc, seq(from= min(dens), to = max(dens), by = 0.1))
lines(x, flexpnr(X= x, b= 0.17, h= 0.14, q= 0.20, T= 24/24), col="grey50", lty=1)
```

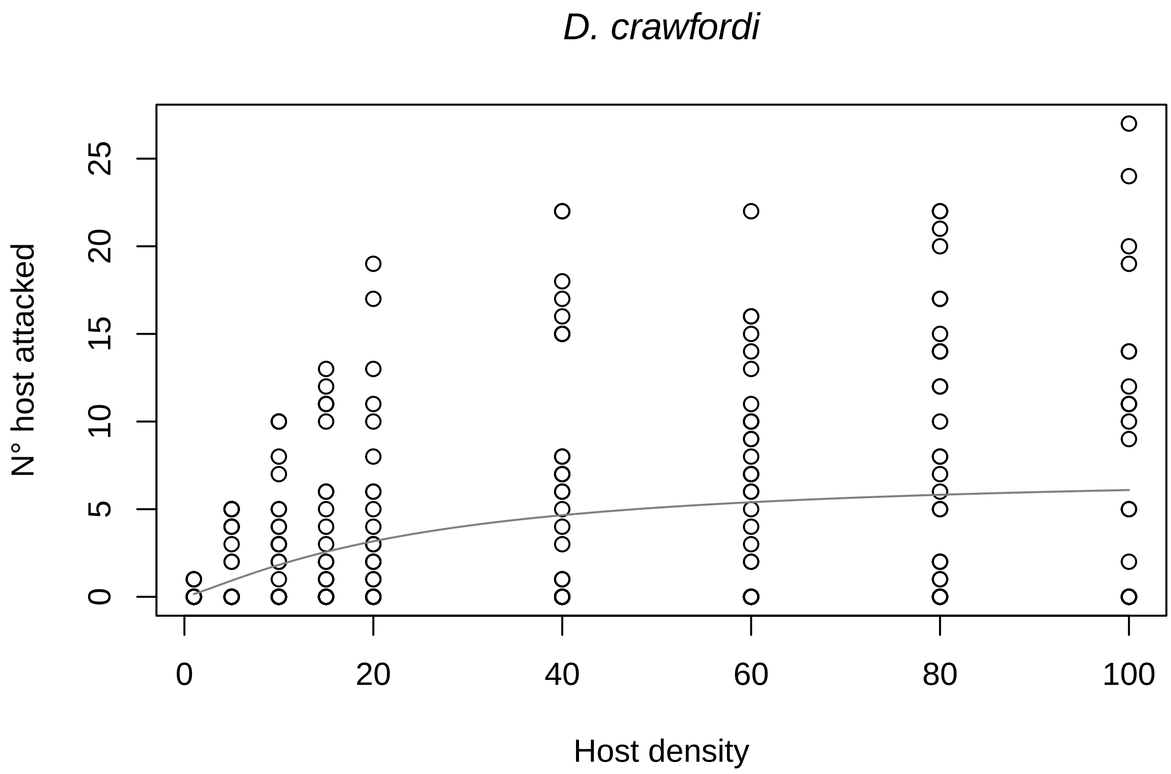

Ganaspis pelleranoi

Variable "q"

```
Gp_flex <- frair_fit(formula = par~dens, data = Gp, response = "flexpnr",
      start = list(b = 1, h = 0.02, q = 0), fixed = list(T = 24/24))

summary(Gp_flex$fit)
```

```
## Maximum likelihood estimation
##
## Call:
## bbmle::mle2(minuslogl = flexpnr_nll, start = start, method = "Nelder-Mead",
##     optimizer = "optim", fixed = fixed, data = list(X = dat$X,
##         Y = dat$Y), control = list(maxit = 5000))
##
## Coefficients:
##    Estimate Std. Error z value     Pr(z)
## b 0.3283299  0.0642361  5.1113   3.2e-07 ***
## q 0.2423913  0.0772540  3.1376  0.001703 **
## h 0.0364548  0.0027906 13.0632 < 2.2e-16 ***
## ---
## Signif. codes:  0 '***' 0.001 '**' 0.01 '*' 0.05 '.' 0.1 ' ' 1
##
## -2 log L: 3225.265
```

```r
Gp_flex$fit
```

```
##
## Call:
## bbmle::mle2(minuslogl = flexpnr_nll, start = start, method = "Nelder-Mead",
##     optimizer = "optim", fixed = fixed, data = list(X = dat$X,
##         Y = dat$Y), control = list(maxit = 5000))
##
## Coefficients:
##          b          q          h          T
## 0.32832990 0.24239125 0.03645481 1.00000000
##
## Log-likelihood: -1612.63
```

"q" fixed to zero:

```r
Gp_II <- frair_fit(formula = par~dens, data = Gp, response = "flexpnr",
      start = list(b = 1, h = 0.02), fixed = list(T = 24/24, q = 0))

summary(Gp_II$fit)
```

```
## Maximum likelihood estimation
##
## Call:
## bbmle::mle2(minuslogl = flexpnr_nll, start = start, method = "Nelder-Mead",
##     optimizer = "optim", fixed = fixed, data = list(X = dat$X,
##         Y = dat$Y), control = list(maxit = 5000))
##
## Coefficients:
##    Estimate Std. Error z value     Pr(z)
## b 0.5873922  0.0281357  20.877 < 2.2e-16 ***
## h 0.0269270  0.0017445  15.435 < 2.2e-16 ***
## ---
## Signif. codes:  0 '***' 0.001 '**' 0.01 '*' 0.05 '.' 0.1 ' ' 1
##
## -2 log L: 3235.82
```

Hassell III

```r
Gp_hass <- frair_fit(formula = par~dens, data = Gp, response = "hassIIInr",
      start = list(b = 0.32, c = 0.02, h = 0.02), fixed = list(T = 24/24))

summary(Gp_hass$fit)
```

```
## Maximum likelihood estimation
##
## Call:
## bbmle::mle2(minuslogl = hassIIInr_nll, start = start, method = "Nelder-Mead",
##     optimizer = "optim", fixed = fixed, data = list(X = dat$X,
##         Y = dat$Y), control = list(maxit = 5000))
##
## Coefficients:
##    Estimate Std. Error z value  Pr(z)
## b 0.5754745  0.3713897  1.5495 0.1213
```

```
## c 0.8762024  0.6246808  1.4026 0.1607
## h 0.0293434  0.0022015 13.3285 <2e-16 ***
## ---
## Signif. codes:  0 '***' 0.001 '**' 0.01 '*' 0.05 '.' 0.1 ' ' 1
##
## -2 log L: 3231.161
```

Model Comparison

```
AIC(Gp_flex$fit, Gp_II$fit, Gp_hass$fit)
```

```
##        AIC df
## 1 3231.265  3
## 2 3239.820  2
## 3 3237.161  3
```

```
AICtab(Gp_flex$fit, Gp_II$fit, Gp_hass$fit)
```

```
##             dAIC df
## Gp_flex$fit 0.0  3
## Gp_hass$fit 5.9  3
## Gp_II$fit   8.6  2
```

Individual plot

```
with(Gp, plot(dens, par, main= c(substitute(paste(italic("G. pelleranoi")))), xlab = "Host density", yla
x <- with(Gp, seq(from= min(dens), to = max(dens), by = 0.1))
lines(x, flexpnr(X= x, b= 0.32, h= 0.03, q= 0.24, T= 24/24), col="grey50", lty=1)
```

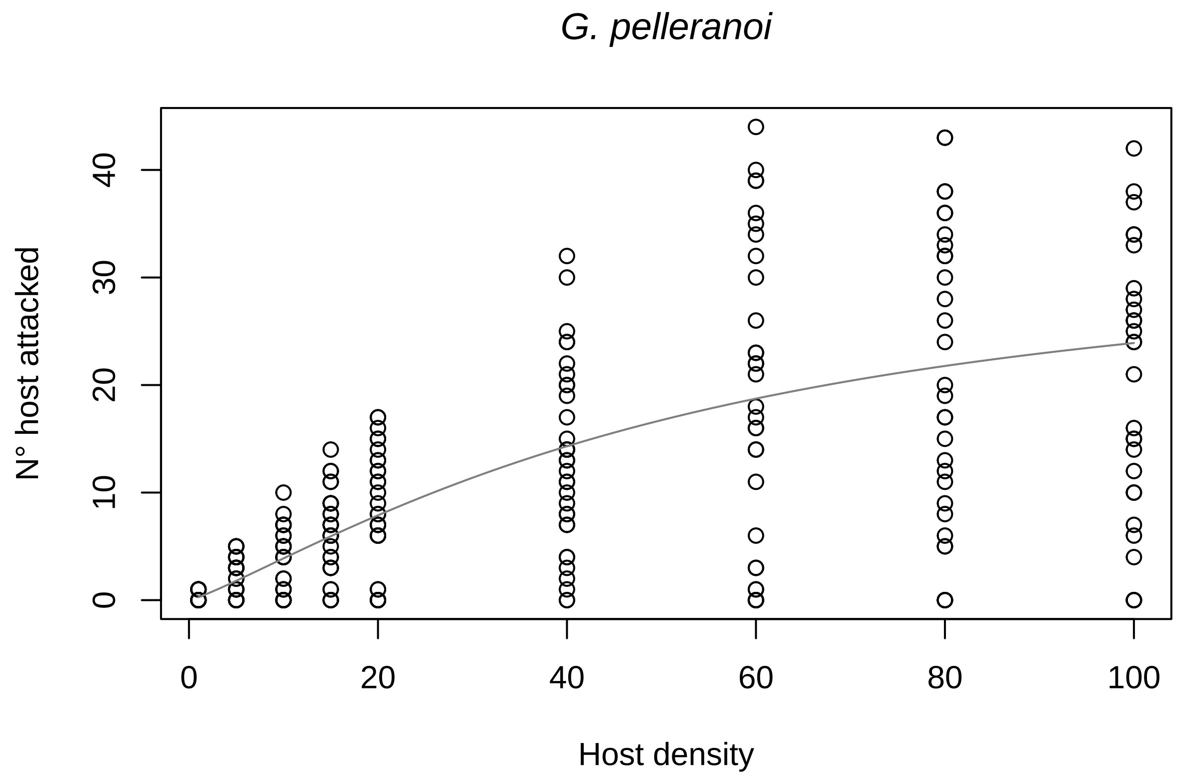

Opius bellus

Variable "q"

```
Ob_flex <- frair_fit(formula = par~dens, data = Ob, response = "flexpnr", start =
                       list(b = 1, h = 0.06, q = 0), fixed = list(T = 24/24))

summary(Ob_flex$fit)
```

```
## Maximum likelihood estimation
##
## Call:
## bbmle::mle2(minuslogl = flexpnr_nll, start = start, method = "Nelder-Mead",
##     optimizer = "optim", fixed = fixed, data = list(X = dat$X,
##         Y = dat$Y), control = list(maxit = 5000))
##
## Coefficients:
##   Estimate Std. Error z value     Pr(z)
## b 0.243384   0.093055  2.6155   0.00891 **
## q 0.069463   0.199115  0.3489   0.72720
## h 0.264182   0.033116  7.9775 1.493e-15 ***
## ---
## Signif. codes:  0 '***' 0.001 '**' 0.01 '*' 0.05 '.' 0.1 ' ' 1
##
## -2 log L: 1343.531
```

"q" fixed to zero:

```
Ob_II <- frair_fit(formula = par~dens, data = Ob, response = "flexpnr", start =
                     list(b = 1, h = 0.06), fixed = list(T = 24/24, q = 0))

summary(Ob_II$fit)
```

```
## Maximum likelihood estimation
##
## Call:
## bbmle::mle2(minuslogl = flexpnr_nll, start = start, method = "Nelder-Mead",
##     optimizer = "optim", fixed = fixed, data = list(X = dat$X,
##         Y = dat$Y), control = list(maxit = 5000))
##
## Coefficients:
##   Estimate Std. Error z value     Pr(z)
## b 0.274106   0.043878  6.2469 4.186e-10 ***
## h 0.254555   0.021163 12.0282 < 2.2e-16 ***
## ---
## Signif. codes:  0 '***' 0.001 '**' 0.01 '*' 0.05 '.' 0.1 ' ' 1
##
## -2 log L: 1343.659
```

Model Comparison

```
AIC(Ob_flex$fit, Ob_II$fit) ##RogersII
```

```
##        AIC df
## 1 1349.531  3
## 2 1347.659  2
```

```
AICtab(Ob_flex$fit, Ob_II$fit)
```

```
##             dAIC df
## Ob_II$fit   0.0  2
## Ob_flex$fit 1.9  3
```

Individual plot

```
with(Ob, plot(dens, par, main= c(substitute(paste(italic("O. bellus")))), xlab = "Host density", ylab =
x <- with(Ob, seq(from= min(dens), to = max(dens), by = 0.1))
lines(x, rogersII(X= x, a= 0.27, h= 0.25, T= 24/24), col="grey50", lty=1)
```

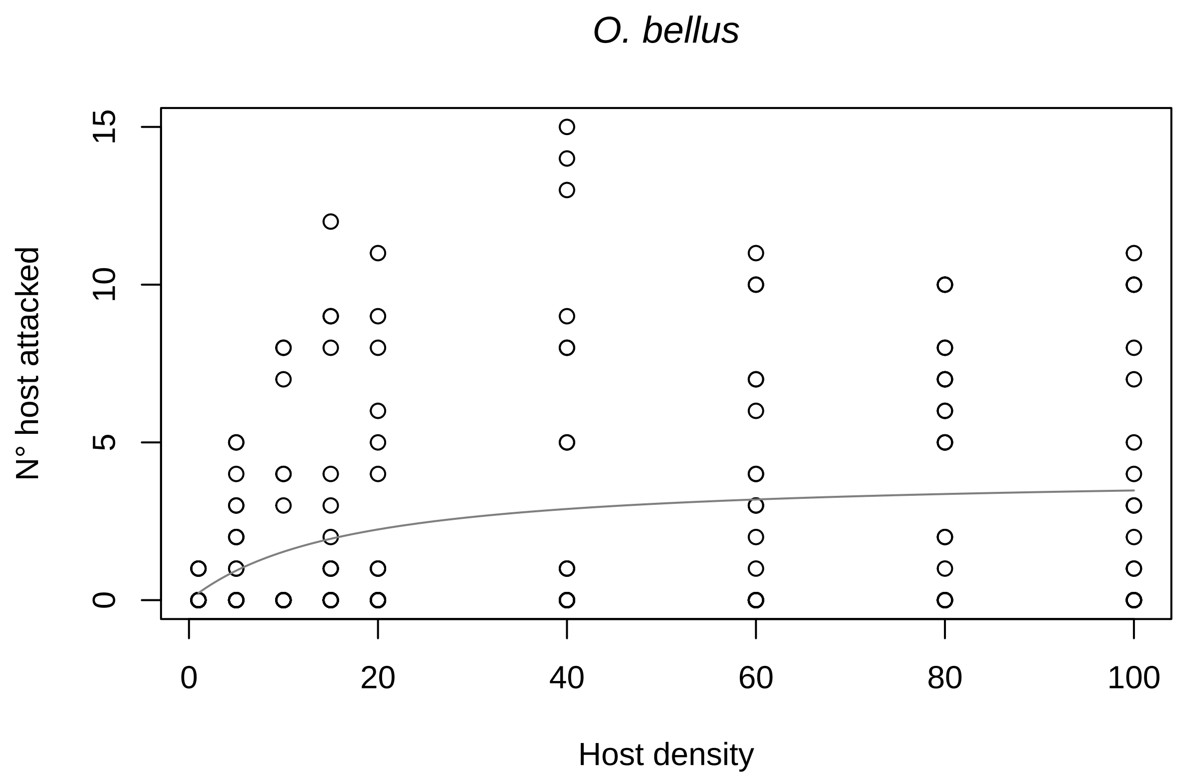

Parameter comparison (frair _compare)

```
st <- list(b= 0.5, h= 0.02, q=0)
st2 <- list(a= 0.5, h= 0.02)
fx <- list(T= 24/24)

ch_fit <- frair_fit(par~dens, data= ch, response = "flexpnr", start = st, fixed = fx)
Ob_fit <- frair_fit(par~dens, data= Ob, response = "rogersII", start = st2, fixed = fx)
Dc_fit <- frair_fit(par~dens, data= Dc, response = "flexpnr", start = st, fixed = fx)
Gp_fit <- frair_fit(par~dens, data= Gp, response = "flexpnr", start = st, fixed = fx)




frair_compare(ch_fit,Dc_fit)
```

```
## FUNCTIONAL RESPONSE COEFFICIENT TEST
##
## Response:            flexpnr
## Optimised variables: b,h,q
## Fixed variables:     T
##
## Original coefficients:
##              b       h       q
## ch_fit 1.55747 0.15292 0.38449
```

```
## Dc_fit 0.17765 0.14981 0.21049
##
## Test: ch_fit - Dc_fit
##
##    Estimate Std. Error z value   Pr(z)
## Db  0.83538    0.30093  2.7760 0.00550 **
## Dq  0.52310    0.25664  2.0382 0.04153 *
## Dh  0.01106    0.01457  0.7588 0.44796
## ---
## Signif. codes:  0 '***' 0.001 '**' 0.01 '*' 0.05 '.' 0.1 ' ' 1
```

```
frair_compare(ch_fit,Gp_fit)
```

```
## FUNCTIONAL RESPONSE COEFFICIENT TEST
##
## Response:            flexpnr
## Optimised variables: b,h,q
## Fixed variables:     T
##
## Original coefficients:
##              b       h       q
## ch_fit 1.55747 0.15292 0.38449
## Gp_fit 0.32876 0.03644 0.24188
##
## Test: ch_fit - Gp_fit
##
##    Estimate Std. Error z value   Pr(z)
## Db  0.10431    0.14991  0.6959 0.48652
## Dq  0.59991    0.17388  3.4501 0.00056 ***
## Dh  0.11740    0.00685 17.1348 < 2e-16 ***
## ---
## Signif. codes:  0 '***' 0.001 '**' 0.01 '*' 0.05 '.' 0.1 ' ' 1
```

```
frair_compare(Dc_fit,Gp_fit)
```

```
## FUNCTIONAL RESPONSE COEFFICIENT TEST
##
## Response:            flexpnr
## Optimised variables: b,h,q
## Fixed variables:     T
##
## Original coefficients:
##              b       h       q
## Dc_fit 0.17765 0.14981 0.21049
## Gp_fit 0.32876 0.03644 0.24188
##
## Test: Dc_fit - Gp_fit
##
##    Estimate Std. Error z value   Pr(z)
## Db -0.17241    0.07888 -2.1857 0.02883 *
## Dq  0.02913    0.15064  0.1934 0.84665
## Dh  0.11817    0.01203  9.8233 < 2e-16 ***
## ---
## Signif. codes:  0 '***' 0.001 '**' 0.01 '*' 0.05 '.' 0.1 ' ' 1
```

Bootstrap, CI and graphics (frair_boot, frair_fit)

```
set.seed(42)
# Bootstrap the G. pelleranoi fit
Gp_fitb <- frair_boot(Gp_fit)
```

```
## BOOTSTRAPPING.
## NB: This function calls the lambertW function. Please be patient.
```

```
confint(Gp_fitb, citypes = 'perc')
```

```
## Coefficient  CI Type        Lower   Upper
## b            Percentile     0.139   0.638
## h            Percentile     0.017   0.049
## q            Percentile     -0.067  0.586
```

```
# Bootstrap the O. bellus fit
Ob_fitb <- frair_boot(Ob_fit)
```

```
## BOOTSTRAPPING.
## NB: This function calls the lambertW function. Please be patient.
```

```
confint(Ob_fitb, citypes = 'perc')
```

```
## Coefficient  CI Type        Lower   Upper
## a            Percentile     0.142   0.511
## h            Percentile     0.169   0.349
```

```
# Bootstrap the D. crawfordi fit
Dc_fitb <- frair_boot(Dc_fit)
```

```
## BOOTSTRAPPING.
## NB: This function calls the lambertW function. Please be patient.
```

```
confint(Dc_fitb, citypes = 'perc')
```

```
## Coefficient  CI Type        Lower   Upper
## b            Percentile     0.048   0.391
## h            Percentile     0.06    0.214
## q            Percentile     -0.236  0.821
```

```
# Bootstrap the C. haywardi fit
ch_fitb <- frair_boot(ch_fit)
```

```
## BOOTSTRAPPING.
## NB: This function calls the lambertW function. Please be patient.
```

```
confint(ch_fitb, citypes = 'perc')
```

```
## Coefficient  CI Type        Lower   Upper
## b            Percentile     0.914   3.127
## h            Percentile     0.114   0.172
## q            Percentile     -0.296  0.686
```

```
### Grafico compartivo entre las especies

old_par <- par()
par(mai=c(0.5, 0.5, 0, 0), omi=c(0.25, 0.25, 0.1, 0.4), las=1)
do_plot(x_axis_labs = TRUE)
```

```
## Warning in data("rf"): data set 'rf' not found
```

```
chc <- brewer.pal(6, "Dark2")[1]
Dcc <- brewer.pal(6, "Dark2")[2]
Gpc <- brewer.pal(6, "Dark2")[3]
Obc <- brewer.pal(6, "Dark2")[6]

drawpoly(ch_fitb, col=adjustcolor(chc, alpha = 0.3), border = chc)
```

```
## Calculating polygons.
```

```
drawpoly(Dc_fitb, col=adjustcolor(Dcc, alpha = 0.3), border = Dcc)
```

```
## Calculating polygons.
```

```
drawpoly(Gp_fitb, col=adjustcolor(Gpc, alpha = 0.3), border = Gpc)
```

```
## Calculating polygons.
```

```
drawpoly(Ob_fitb, col=adjustcolor(Obc, alpha = 0.3), border = Obc)
```

```
## Calculating polygons.
```

```
lines(ch_fitb, col=chc)
lines(Dc_fitb, col=Dcc)
lines(Gp_fitb, col=Gpc)
lines(Ob_fitb, col=Obc)
```

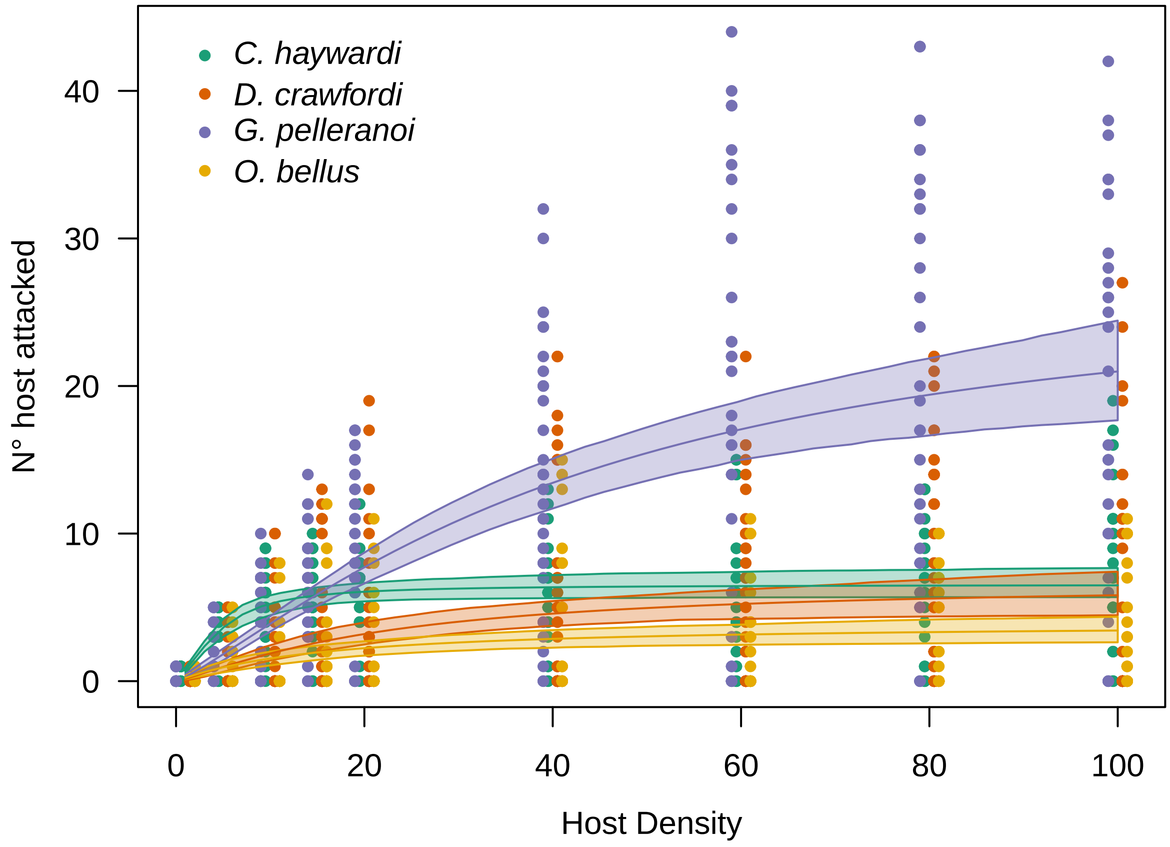


```
layout(matrix(1)); par <- old_par
```

Individual plots joined

```r
### Species individual plots

old_par <- par()
par(mfrow = c(2, 2)) ###Creo un espacio para 4 gráficos juntos
par(mai=c(0.8, 0.8, 0, 0), omi=c(0.25, 0.25, 0.1, 0.4), las=1)

# plot individual Gp
do_Gp_plot(x_axis_labs = F)
```

```
## Warning in data("Gp"): data set 'Gp' not found
```

```r
Gpc <- brewer.pal(4, "Dark2")[3]
drawpoly(Gp_fitb, col=adjustcolor(Gpc, alpha = 0.3), border = Gpc)
```

```
## Calculating polygons.
```

```r
lines(Gp_fitb, col=Gpc)

# plot individual Ob
do_Ob_plot(x_axis_labs = F)
```

```
## Warning in data("Ob"): data set 'Ob' not found
```

```r
Obc <- brewer.pal(4, "Dark2")[3]
drawpoly(Ob_fitb, col=adjustcolor(Obc, alpha = 0.3), border = Obc)
```

```
## Calculating polygons.
```

```r
lines(Ob_fitb, col=Obc)
# plot individual Dc
do_Dc_plot(x_axis_labs = TRUE)
```

```
## Warning in data("Dc"): data set 'Dc' not found
```

```r
Dcc <- brewer.pal(4, "Dark2")[3]
drawpoly(Dc_fitb, col=adjustcolor(Dcc, alpha = 0.3), border = Dcc)
```

```
## Calculating polygons.
```

```r
lines(Dc_fitb, col=Dcc)
# plot individual Ch
do_Ch_plot(x_axis_labs = TRUE)
```

```
## Warning in data("ch"): data set 'ch' not found
```

```r
chc <- brewer.pal(4, "Dark2")[3]
drawpoly(ch_fitb, col=adjustcolor(chc, alpha = 0.3), border = chc)
```

```
## Calculating polygons.
```

```r
lines(ch_fitb, col=chc)
```

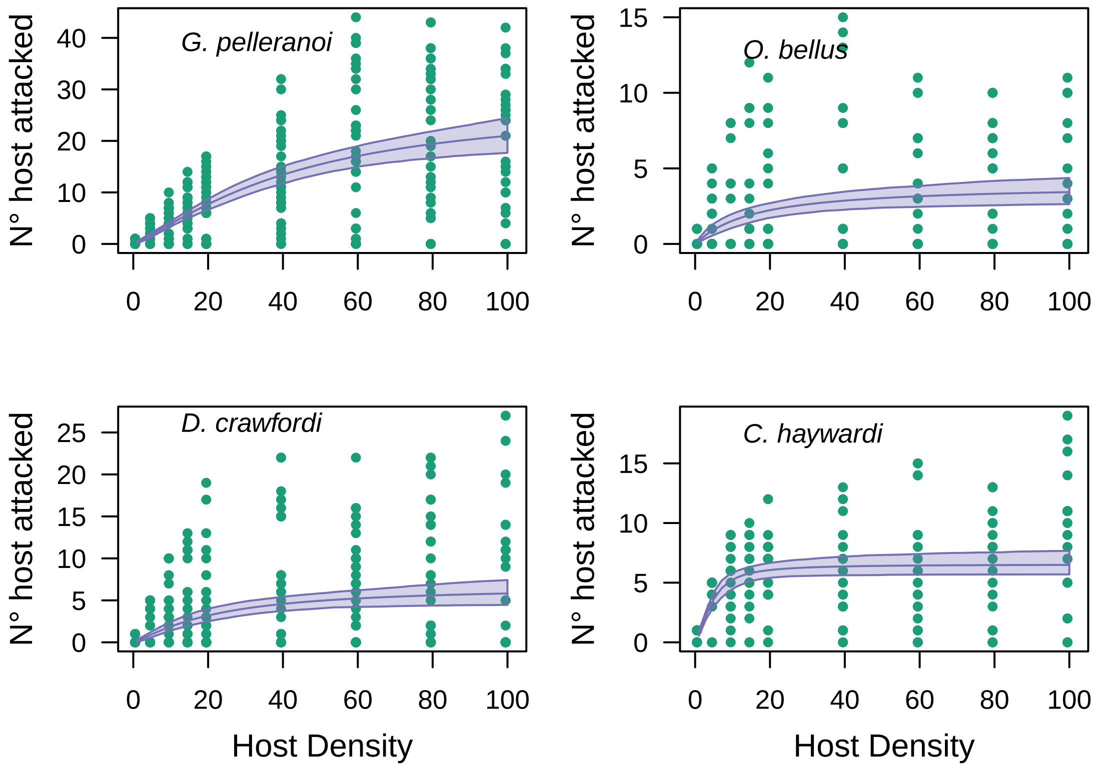


```
par(mfrow = c(1, 1))
```

Expected values of host attacked

Expected number of host attacked considering each experimental density, from “flexpnr” and “rogersII” functions of the Frair packages. The values of b, h y q were obtained above (ANTERNATIVE METHOD).

```
ch_values <- flexpnr(X= c(1, 5, 10, 15, 20, 40, 60, 80, 100), b= 1.555, h= 0.153, q=0.386, T= 24/24)
dc_values <- flexpnr(X= c(1, 5, 10, 15, 20, 40, 60, 80, 100), b= 0.17, h= 0.14, q= 0.20, T= 24/24)
gp_values <- flexpnr(X= c(1, 5, 10, 15, 20, 40, 60, 80, 100), b= 0.32, h= 0.03, q= 0.24, T= 24/24)
ob_values <- rogersII(X= c(1, 5, 10, 15, 20, 40, 60, 80, 100), a= 0.27, h= 0.25, T= 24/24)

Exp_values <- data.frame(
  "Density"= c(1, 5, 10, 15, 20, 40, 60, 80, 100),
  "Ch_val" = ch_values,
  "Dc_val" = dc_values,
  "Gp_val" = gp_values,
  "Ob_val" = ob_values)

Exp_values %>%
  kbl(caption = "Expected number of host attacked by each density") %>%
  kable_classic(full_width = F, html_font = "Cambria")
```


Pritchard, Daniel W., Rachel A. Paterson, Helene C. Bovy, and Daniel Barrios-O’Neill. 2017. “Frair : An R Package for Fitting and Comparing Consumer Functional Responses.” Edited by Timothée Poisot. *Methods in Ecology and Evolution* 8 (11): 1528–34. https://doi.org/10.1111/2041-210x.12784.

Table 1: Expected number of host attacked by each density

Density	Ch_val	Dc_val	Gp_val	Ob_val
1	0.7476939	0.1532524	0.2719527	0.2249413
5	3.6233182	0.9236058	1.7974203	0.9345640
10	5.2495986	1.8192978	3.8819081	1.5336124
15	5.8116690	2.5627764	5.9363178	1.9438445
20	6.0587045	3.1658377	7.8860222	2.2401330
40	6.3606845	4.6563349	14.3078129	2.8892802
60	6.4377263	5.3967242	18.7363188	3.1905030
80	6.4706305	5.8214202	21.7729471	3.3636313
100	6.4882834	6.0915553	23.9086609	3.4758908
